# Promotion of a cancer-like phenotype, through chronic exposure to inflammatory cytokines and hypoxia in a bronchial epithelial cell line model

**DOI:** 10.1038/srep18907

**Published:** 2016-01-13

**Authors:** Anne-Marie Baird, Steven G. Gray, Derek J. Richard, Kenneth J. O’Byrne

**Affiliations:** 1 Cancer and Ageing Research Program, Queensland University of Technology, Brisbane, Australia; 2 Thoracic Oncology Research Group, Institute of Molecular Medicine, Trinity College Dublin, Ireland; 3 HOPE Directorate, St. James’s Hospital, Dublin 8, Ireland; 4 Division of Cancer Services, Princess Alexandra Hospital, Brisbane, Australia

## Abstract

Globally, lung cancer accounts for approximately 20% of all cancer related deaths. Five-year survival is poor and rates have remained unchanged for the past four decades. There is an urgent need to identify markers of lung carcinogenesis and new targets for therapy. Given the recent successes of immune modulators in cancer therapy and the improved understanding of immune evasion by tumours, we sought to determine the carcinogenic impact of chronic TNF-α and IL-1β exposure in a normal bronchial epithelial cell line model. Following three months of culture in a chronic inflammatory environment under conditions of normoxia and hypoxia (0.5% oxygen), normal cells developed a number of key genotypic and phenotypic alterations. Important cellular features such as the proliferative, adhesive and invasive capacity of the normal cells were significantly amplified. In addition, gene expression profiles were altered in pathways associated with apoptosis, angiogenesis and invasion. The data generated in this study provides support that TNF-α, IL-1β and hypoxia promotes a neoplastic phenotype in normal bronchial epithelial cells. In turn these mediators may be of benefit for biomarker and/or immune-therapy target studies. This project provides an important inflammatory *in vitro* model for further immuno-oncology studies in the lung cancer setting.

Lung cancer is the leading cause of cancer related death in both men and women, accounting for nearly one in five cancer deaths^1^. In 2012, 1.59 million deaths were attributed to this disease^2^. Five-year survival rates continue to remain poor at approximately 15%. A number of factors play a role in the etiology of lung cancer including smoking, radon and a number of environmental pollutants^3^. These factors can result in inflammation within the lung. Inflammation is a crucial part of the innate immune system that protects against pathogens and initiates adaptive immunity. Acute inflammation is usually a rapid and self-limiting process, however it does not always resolve. This leads to the establishment of a chronic inflammatory state.

It is acknowledged that chronic inflammation plays an important role in cancer initiation and progression in a variety of solid cancers^4^,^5^,^6^,^7^. This includes lung cancer^8^,^9^,^10^,^11^, as individuals with inflammatory lung conditions are at an increased risk of lung cancer development even in the absence of tobacco use^10^. Additionally, elevated scores in inflammatory based indices such as neutrophil-to-lymphocyte ratio (NLR)^12^ and Glasgow Prognostic Score (GPS)^13^ can signify poor prognosis and survival in this disease. Furthermore, a number of studies have demonstrated an association with small nucleotide polymorphisms (SNPs) in inflammatory genes with an increased risk of non-small cell lung cancer (NSCLC), including Tumour Necrosis Factor alpha (TNF-α)^14^ and Interleukin-1 beta (IL-1β)^15^. However, the relationship between SNPs and lung cancer risk appears to be somewhat dependent on ethnicity. TNF-α and IL-1β are often referred to as ‘alarm’ cytokines as they play critical roles in immune and inflammatory responses.

TNF-α is involved in normal physiological processes such as proliferation, apoptosis and differentiation, however, it can play a pathophysiological role when it becomes deregulated^16^. It is linked to a number of carcinogenic activities such as invasion, angiogenesis, proliferation and transformation^17^. TNF-α serum levels are higher in lung cancer cases compared with controls^18^,^19^,^20^,^21^,^22^, levels increase with stage and smoking status^20^ and are associated with a worse prognosis^22^. Moreover, the expression of seventeen TNF-α mediated genes can predict recurrence free survival in the disease^23^, while levels of this cytokine can serve as a predictive marker to chemotherapy treatment^24^.

IL-1β primarily affects inflammation and immune responses, but also regulates homeostatic functions^25^. Similar to TNF-α, deregulation of IL-1β can have pathological consequences. In cancer it is linked to neoplastic transformation, angiogenesis, tumour invasiveness and metastasis^25^. In lung cancer, IL-1β serum concentrations are significantly higher in lung cancer patients compared with controls and levels are associated with a worse prognosis^22^. High levels of this cytokine are linked to shorter progression free survival and overall survival in the disease^26^.

A common feature of inflamed tissues is a central hypoxic core, similar to that found in the central mass of solid tumours, with both inflammation and hypoxia fuelling each other^27^. Both conditions also promote NF-ĸB, resulting in the up regulation of TNF-α, IL-1β and Hypoxia Inducible Factor 1 alpha (HIF1-α)^27^,^28^. HIF1-α is a critical mediator of the hypoxic response, which can alter the expression of over 60 genes linked to immune response, invasion, metastases and resistance to apoptosis^29^. In lung cancer, HIF1-α was identified as part of a three-gene signature, which could classify early stage NSCLC patients with difference prognoses^30^. Hypoxia surrogacy markers such as carbonic anhydrase (CA) IX are associated with a poor prognosis when overexpressed in NSCLC^31^.

This study sought to determine the effect of chronic TNF-α and IL-1β exposure, alone or in combination, in a normal cell line, cultured under conditions of normoxia and hypoxia (0.5% oxygen). The cell line chosen was the human bronchial epithelial cell line, HBEC4. This cell line was immortalised in the absence of viral onco-proteins, by the over-expression of CDK4 to abrogate the p16/Retinoblastoma (Rb) pathway, and by the expression of ectopic human telomerase reverse transcriptase (hTERT) to bypass replicative senescence^32^. While these cells are clonable, they do not form colonies in soft agar or tumours in nude mice^32^. In addition, HBECs grown in a 3D tissue model retained their ability to differentiate into basal, mucin producing, and columnar ciliated epithelial cells^33^. However, malignancy was conferred on these cells by three additional genetic changes consisting of c-Myc over-expression, increase in KRAS^V12^ and p53 knockdown^34^. The cell donor (HBEC4) subsequently developed lung cancer^32^ and we hypothesised that this predisposition of the donor epithelium to develop lung cancer combined with chronic inflammatory and hypoxia exposure, may promote malignant transformation in the HBEC4 cells.

The immuno-oncology field is rapidly progressing in terms of targeted immune therapies and elucidation of tumour immune evasion. This project provides an important inflammatory *in vitro* model for further immuno-oncology studies and may be of benefit for immune based biomarker studies to augment lung cancer screening protocols and enhance our knowledge of inflammatory linked lung carcinogenesis pathways.

## Results

### HBEC4 cell lines

The HBEC4 cells were stably transfected with TNF-α and IL-1β alone or in combination, creating four sub-lines (a) Empty vector control (EVC), (b) TNF-α alone, (c) IL-1β alone and (d) TNF-α and IL-1β combined. Un-transfected HBEC4 cell lines were also used and are referred to as HBEC4 ctl. Cells were cultured under normal oxygen conditions (Normoxia) and 0.5% oxygen (Hypoxia). To confirm cells were experiencing hypoxic conditions, pO_2_ measurements were recorded by means of a pO_2_ probe (Supplementary Fig. 1). A number of cellular characteristics were examined at the beginning of the long-term experiment and are referred to as ‘Time 0’, while experiments completed after three months of continuous culture are referred to as ‘Time 3’.

### TNF-α and IL-1β mRNA and protein levels alter over time

#### Morphology

**Figure 1:**
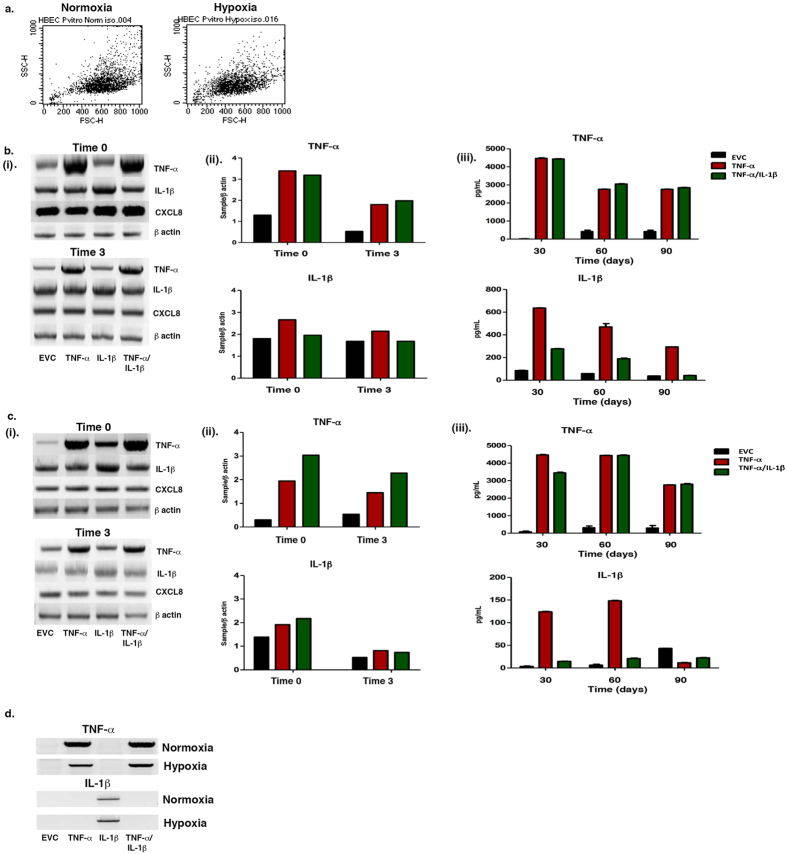
Three months of culture resulted in adaptations to cellular size and the expression of cytokines at both the mRNA and protein level. (**a**) Cells cultured under hypoxic conditions were smaller than those under normoxia as observed by FACS. Normoxic cells group at approximately 400–600 compared with the hypoxic cells, which group at approximately 250–600. The FSC-H label refers to the light scatter in the forward direction. (Dot plot is representative of normoxic and hypoxic cells: 20,000 events, n = 1). (**b**) Expression of TNF-α, IL-1β and CXCL8 in clones cultured under normoxia. Cytokine expression at the (i) mRNA level with (ii) densitometry analysis, and (iii) secreted protein levels in early (Time 0) and three month old clones (Time 3). mRNA values are expressed as a ratio of target gene band intensity: β actin band intensity (loading control). Secreted cytokine values are per 1 × 10^6^ cells seeded. Samples were assayed in triplicate and data is expressed as mean ± SEM. (**c**) Expression of TNF-α, IL-1β and CXCL8 in clones cultured under hypoxia. Cytokine expression at the (i) mRNA level with (ii) densitometry analysis, and (iii) secreted protein levels in early (Time 0) and three month old clones (Time 3). mRNA values are expressed as a ratio of target gene band intensity: β actin band intensity (loading control). Secreted cytokine values are per 1 × 10^6 ^cells seeded. Samples were assayed in triplicate and data is expressed as mean ± SEM. (**d**) Presence of TNF-α and IL-1β inserts in the genomic DNA isolated from HBEC4 clones (as indicated). DNA was isolated from each clone, which were cultured under normoxia and hypoxia for three months. A PCR was carried out for TNF-α and IL-1β.

Initially, growth rates were slower under hypoxia compared with normoxia as cells adapted to the environment. Cellular morphology did not change over time, however hypoxic cells were smaller in size as determined by the forward light scatter using FACS (Fig. 1A). This alteration may be due to a modification in cell spread or variants in the attachment to the collagen matrix. Hypoxia has been shown to influence the shape of cells such as macrophages^35^.

#### Normoxia

The mRNA and protein expression levels of both cytokines were determined at the beginning (Time 0) and end (Time 3) of the experiment (Fig. 1B). *CXCL8* expression was included as a positive control, as both TNF-α and IL-1β affect the expression of this chemokine (Fig. 1Bi). Under normoxia, over the three-month period, *TNF-α* expression showed an almost 2-fold decrease in the TNF-α clone (3.39 *vs.* 1.79) and a similar decrease in the TNF-α/IL-1β clone (3.19 *vs.* 1.97) (Fig. 1Bii). *IL-1β* analysis demonstrated a slight decrease in the IL-1β (2.66 *vs.* 2.19) and TNF-α/IL-1β (1.95 *vs.* 1.68) clone (Fig. 1Bii). In terms of protein secretion, TNF-α decreased from 4465.04 ± 26.81 pg/mL after one month of culture, to 2754.88 ± 12.94 pg/mL after three months (Fig. 1Biii). IL-1β protein levels also decreased with time (637.09 ± 1.59 pg/mL *vs*. 294.48 ± 0.64 pg/mL, Time 0 *vs*. Time 3 respectively) (Fig. 1Biii). IL-1β levels decreased in the TNF-α/IL-1β clone; 276.79 ± 3.15 pg/mL to 42.48 ± 0.54 pg/mL (Fig. 1Biii).

#### Hypoxia

Under hypoxia, *TNF-α* expression remained stable in the TNF-α clone (Fig. 1Ci). However, it reduced by 1.33 fold in the TNF-α/IL-1β clone (3.03 *vs.* 2.27) (Fig. 1Cii). Larger decreases were evident for *IL-1β* in the IL-1β clone, of over 2-fold (1.91 *vs.* 0.81) and TNF-α/IL-1β clone of over 3-fold (2.17 *vs.* 0.73) (Fig. 1Cii). TNF-α secretion also reduced under hypoxia from one to three months (Fig. 1Ciii); TNF-α clone: 4465.04 ± 26.81 pg/mL *vs.* 2799.13 ± 31.43 pg/mL, TNF-α/IL-1β clone: 3445.58 ± 37.73 pg/mL *vs.* 2799 ± 31.43 pg/mL. The decline in IL-1β levels was more pronounced at three months (Fig. 1Ciii).

#### Decrease evident in protein and mRNA levels of both cytokines after three months

The reductions observed over time may be attributed a number of issues such as negative feed back loops caused by the induction of other cytokines or chemokines particularly under hypoxic conditions. However, the examination of genomic DNA for the presence or absence of the TNF-α/IL-1β, demonstrated that IL-1β expression was absent from the TNF-α/IL-1β clone (Fig. 1D). This suggests that a recombination event had taken place within the IL-1β gene in the TNF-α/IL-1β clone, which prevented IL-1β over-expression.

### Genes involved in the hallmarks of cancer are modified in response to chronic inflammation and hypoxia

**Figure 2:**
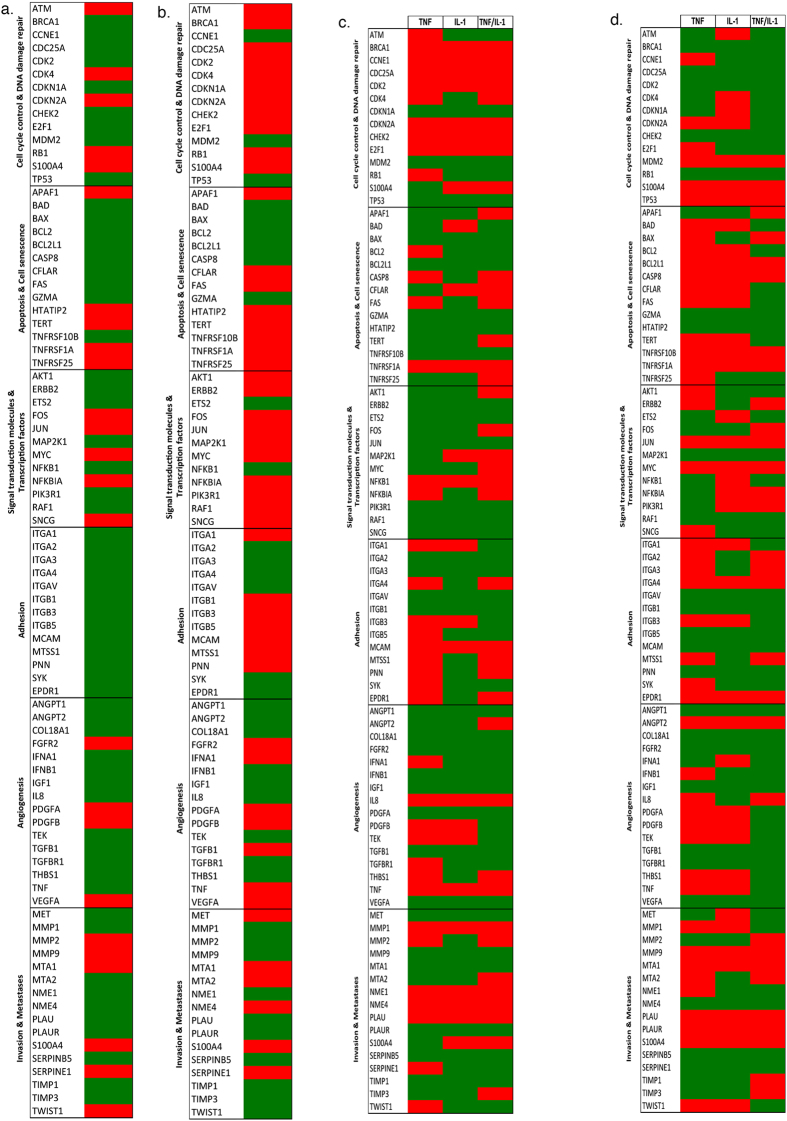
Exposure to inflammatory mediators and hypoxia alters the gene expression profile of HBEC4. Heat maps generated from data obtained from the Human Cancer PathwayFinder™ RT^2^ Profiler (**a**) hypoxic HBEC4 ctl *vs.* normoxic HBEC4 ctl, (**b**) hypoxia EVC *vs.* normoxia EVC, (**c**) normoxia overexpression clones *vs*. Normoxia EVC clone, (**d**) hypoxia overexpression clones *vs*. hypoxia EVC clone. Clones were cultured for three months. (Red shading specifies a gene that is up-regulated and green shading indicates a gene that is down-regulated).

Chronic inflammation and cancer are linked in part through the ability of inflammation to aid in the transformation of a pre-neoplastic cell into a malignant phenotype via the promotion of the hallmarks of cancer. After three months of culture cell lines were analysed using the Cancer PathwayFinder^™^ Array. Fold changes were determined by comparing (i) Hypoxia HBEC4 ctl *vs.* Normoxia HBEc4 ctl (Fig. 2A), (ii) Hypoxia EVC *vs.* Normoxia EVC (Fig. 2B), (iii) Normoxia over-expression clones *vs.* Normoxia EVC (Fig. 2C) and (iv) Hypoxia overexpression clones *vs.* hypoxic EVC (Fig. 2D).

Over 28% (24/84) of genes were up regulated in the HBEC4 ctl cultured under hypoxic compared with normoxia (Fig. 2A), while the EVC, displayed an increase in 57% (48/84) of genes under hypoxic conditions (Fig. 2B). This included *S100A4* and *VEGFA* in both. There was an increase in *MMP1* in all normoxia overexpression clones and a decrease in *TIMP-1*. *ITGA4* was up regulated in the TNF-α and TNF-α/IL-1β clones, while *ITGA1* was increased in the IL-1β clone (Clones *vs*. EVC) (Fig. 2C). There was an up-regulation of *S100A4*, *MMP9* and *ITGA4* in the hypoxic overexpression clones (Fig. 2D).

A number of genes were validated using qPCR (Supplementary Fig. 2). *S100A4* is important in the metastatic potential of cells^36^ and was increased under hypoxia in both the HBEC4 ctl, EVC and overexpression clones in normoxia and hypoxia. The expression levels of *c-Myc* and *p53* were also chosen for validation, as these genes were part of a five gene change in a normal bronchial epithelial cell, which resulted in malignant transformation^37^. As outlined in Supplementary Fig. 2, *p53* levels were reduced in hypoxia in the HBEC4 ctl cells. The expression varied for the overexpression clones with *p53* levels up regulated in the TNF-α and IL-1β clones under normoxia and down regulated under hypoxia (Supplementary Fig. 2). While c-Myc levels were increased in these overexpression clones under normoxia and hypoxia. It should be noted however, that none of the fold changes were above two (when compared with the appropriate control).

### TNF-α, IL-1β and hypoxia increase cellular proliferation

**Figure 3:**
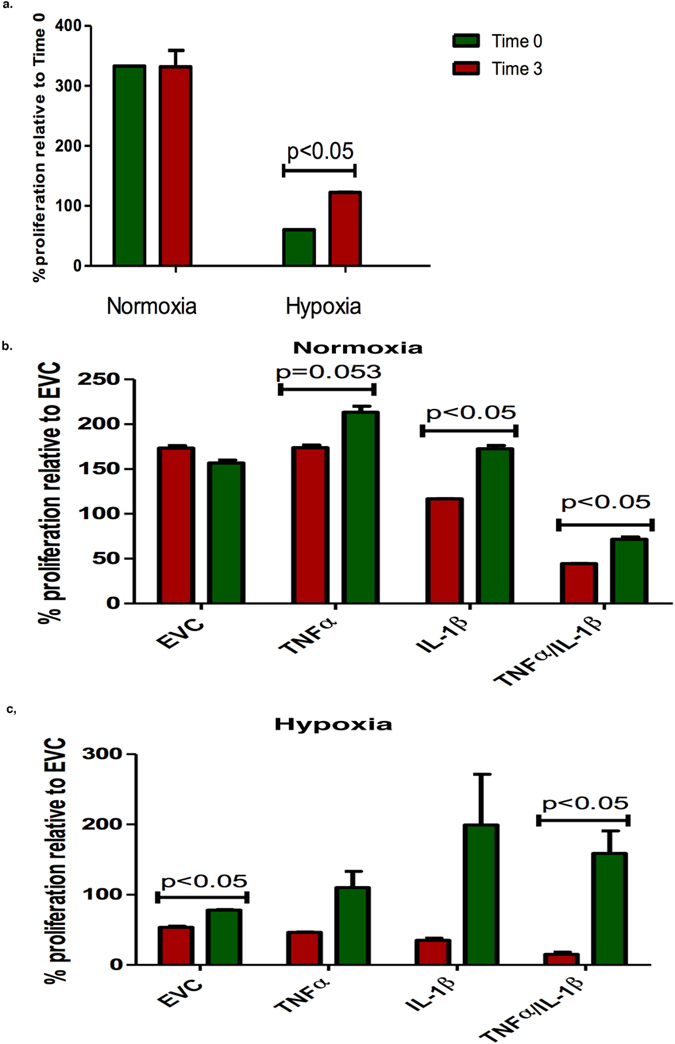
Inflammatory mediators and hypoxia increase cellular proliferation. A comparison of proliferation at Time 3 with Time 0 was examined in (**a**) HBEC4 ctl (**b**) normoxic clones and (**c**) and hypoxic clones. (Data is expressed as mean ± SEM, n = 3, Statistical analysis based on a paired student’s *t* test: time 3 *vs.* time 0).

A cell proliferation BrdU ELISA was utilised to examine the effect of TNF-α, IL-1β and hypoxia over time. Cells were cultured for 24 and 48 h. The normoxia EVC and HBEC4 ctl at 24 h was set as 100% (as appropriate) and all clones at 48 h were normalised to their applicable controls (Fig. 3).

After three months of culture the proliferative rate increased significantly (p < 0.05) under hypoxia for both the HBEC4 ctl (Fig. 3A) and the EVC (Fig. 3C) when compared with time 0. As expected no differences in the proliferation rate of the HBEC4 ctl and EVC were observed under normoxia. All over-expression clones developed an increased proliferative capacity after three months under normoxia (Fig. 3B) (IL-1β, TNF-α/IL-1β - Time 3 *vs.* Time 0, p < 0.05). A similar trend was observed under hypoxic conditions (Fig. 3C) (EVC, TNF-α/IL-1β – Time 3 *vs.* Time 0, p < 0.05). This indicates that as the cells became accustomed to the chronic inflammatory and hypoxic conditions, the proliferative rate increased over time (Time 3 *vs.* Time 0).

### Migration potential is influenced by oxygen conditions

To examine the migration potential of the cells after three months of growth under normoxic and hypoxic conditions, a scratch assay was performed.

**Figure 4:**
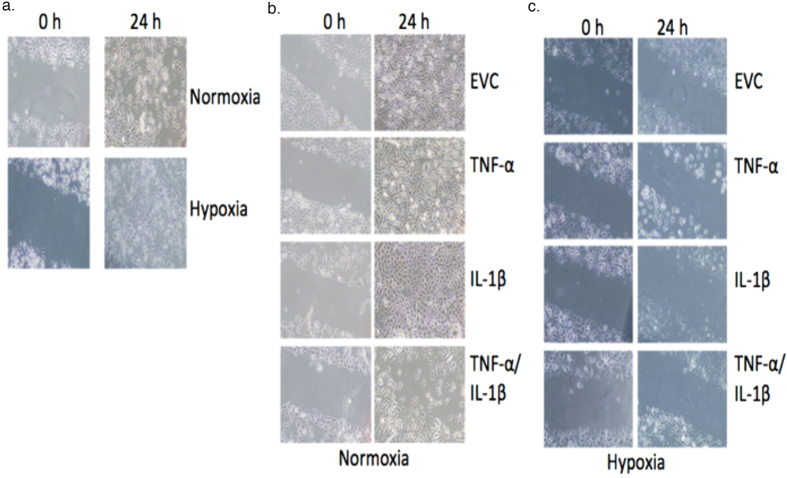
Migration capacity is inhibited under hypoxia. A qualitative scratch assay was performed on HBEC4 ctl and overexpression clones under normoxia and hypoxia. The HBEC4 ctl and over-expression clones were grown to confluence and scratched with a sterile p200 pipette tip. Images were taken at time 0 and 24 h on (**a**) HBEC4 ctl, (**b**) normoxic clones and (**c**) and hypoxic clones. Images are representative of three independent experiments.

Qualitatively, HBEC4 ctl began to close the scratch at 12 h under hypoxia (Data not shown) and by 24 h the scratch was fully closed under both conditions (Fig. 4A). The EVC, TNF-α, IL-1β and TNF-α/IL-1β clones closed the wound after 24 h under normoxia (Fig. 4B); however, they were unable to do so under hypoxia (Fig. 4C). This result would suggest that hypoxia interferes with the clones’ ability to migrate and close the wound, possibly through the up-regulation of adhesion or other molecules. Also, perhaps the integration of the plasmid DNA in these clones affected certain genes, as the HBEC4 ctl had the ability to close the scratch under both oxygen conditions compared with the EVC.

### ICAM and VCAM levels are altered by both inflammatory and hypoxia exposure

**Figure 5:**
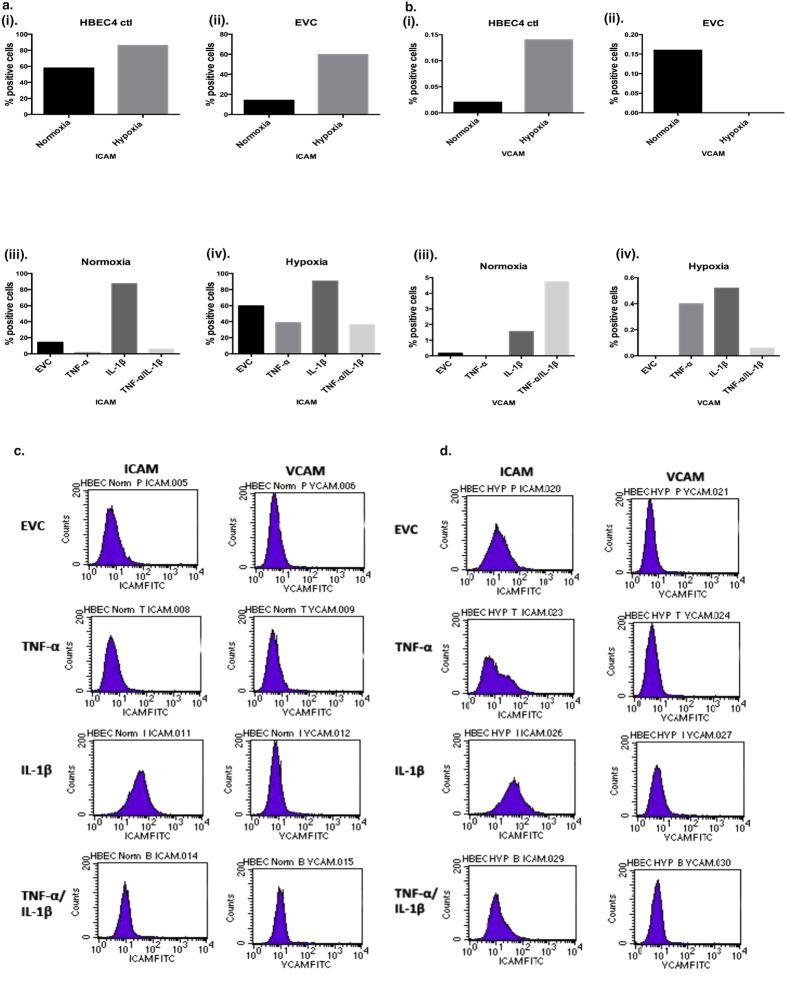
Adhesion molecule expression is altered due to chromic inflammatory and hypoxia exposure. Cell surface staining of (**a**) ICAM-1, (**b**) VCAM-1 by FACS on (i) HBEC4 ctl, (ii) EVC clones, (iii) normoxia clones and (iv) hypoxia clones and representative flow cytometry plots for (**c**) Normoxia and (**d**) Hypoxia. Cells were continuously cultured for a period of three months. Values based on a dot blot analysis using percentage-gated values. Isogenic controls were used for each individual sample and this value was subtracted from each sample value. (20,000 events counted, n = 1).

The capacity of cells to close a wound depends on a number of factors, particularly adhesion molecules such as ICAM-1 and VCAM-1. These adhesion molecules can be regulated by TNF-α and IL-1β^38^. In the HBEC4 ctl there was 1.5 fold increase in expression of ICAM (Hypoxia *vs*. Normoxia) (Fig. 5Ai), this increase was also observed in the EVC (59.77% *vs.* 14.23%) (Fig. 5Aii). Between overexpression clones, IL-1β induced ICAM expression under both normoxic (87.27% *vs.* 14.23%) and hypoxic conditions (90.44% *vs.* 59.77%) when compared with the EVC (Fig. 5Aiii/iv). Levels of VCAM-1 were much lower than ICAM-1, however increases in expression were observed in the IL-1β and TNF-α/IL-1β (normoxia) and TNF-α and IL-1β clones (hypoxia) (Fig. 5Biii/iv). It is not unexpected than VCAM-1 levels are lower, as this molecule would not ordinarily be expressed on epithelial cells.

### Hypoxia and dual exposure to TNF-α and IL-1β significantly enhances cellular invasive capacity

**Figure 6:**
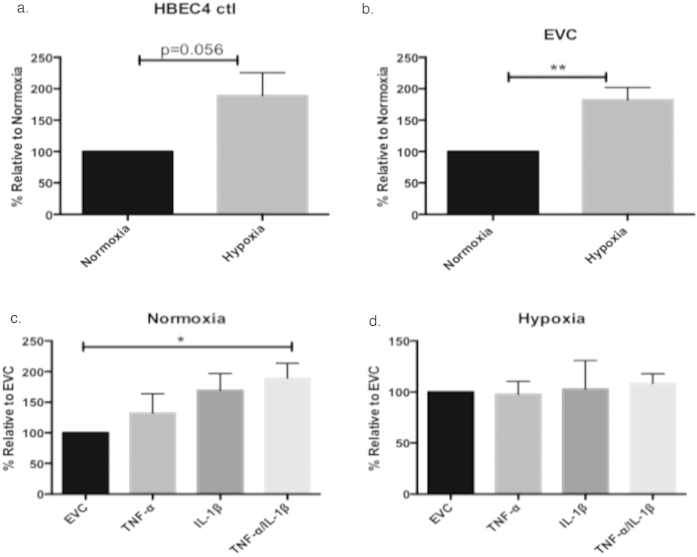
Effects of chronic exposure to hypoxic and/or TNF-α/IL-1β on cellular invasive capacity. The invasive capacity was examined using a florescent-based kit after three months of continuous culture (**a**) HBEC4 ctl, (**b**) EVC, (**c**) normoxic clones and (**d**) hypoxic clones. Data is expressed as percentage relative to appropriate controls and graphed as mean ± SEM (n = 6). (** p < 0.01, paired two-tailed student’s *t* test; *p < 0.05, a one-way ANOVA with Dunnett’s multiple comparisons test).

There was an increase in the invasive capacity of the HBEC4 ctl line (Fig. 6A, p = 0.056) and a significant increase in the EVC control (Fig. 6B) under hypoxic conditions when compared with normoxia (p < 0.001, EVC - Hypoxia 182.3% ± 19.7% *vs.* 100%). Under normoxia, there was an elevation in the invasion capacity of the over-expression clones compared with EVC. This was significant for the TNF-α/IL-1β clone (p < 0.01, 189.4% ± 24.3% *vs.* 100%) (Fig. 6C). There were no differences observed between the hypoxic over-expression clones compared with the hypoxic EVC (Fig. 6D). These results would suggest that the combined effect IL-1β and TNF-α is a potent promoter of invasive cellular capacity under normoxia. However, chronic inflammation does not seem to provide an additive effect in low oxygen conditions.

### Angiogenic secretome profile is differentially modified through inflammatory and hypoxia exposure

**Figure 7:**
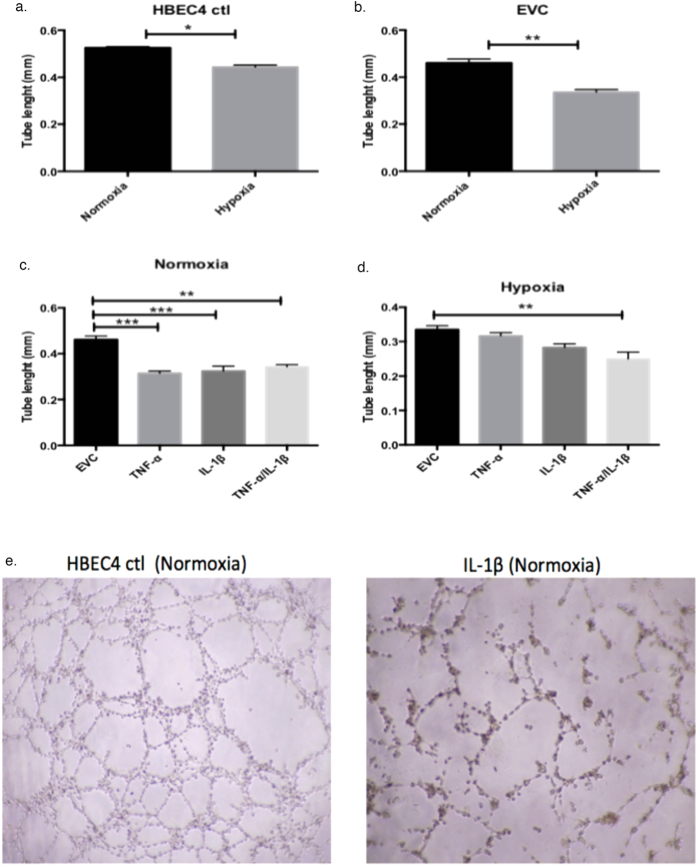
Chronic secretion of inflammatory mediators results in impaired endothelial tube formation. Results are presented graphically for (**a**) HBEC4 ctl, (**b**) EVC, (**c**) normoxia clones and (**d**) hypoxia clones. Representative images from the endothelial tube formation assay are shown in (**e**). Thirty random tube lengths were quantified within the field of view using Image J software. Data is expressed at men ± SEM (n = 3). Images are representative of three independent experiments (*p < 0.05, **p < 0.01, ***p < 0.001–based on two-tailed student’s *t* test (groups of two) and a one-way ANOVA with Dunnett’s multiple comparisons test (groups greater than two)).

TNF-α and IL-1β are involved in both pro- and anti-angiogenic effects, depending upon the concentration of cytokines. To determine if the HBEC4 ctl and the over-expression clones had an altered angiogenic profile after three months of growth, an endothelial tube formation assay was performed. Ea.hy926 cells were seeded on Matrigel in supernatant collected from the HBEC4 ctl and the over-expression clones under normoxia and hypoxia. The cells were incubated in the supernatant and the tube formation quantified after 24 h (Fig. 7A–D). Figure 7E shows representative images obtained after 24 h.

In the HBEC4 ctl, there was a significant decrease in tube length under hypoxia compared with normoxia (p < 0.05, 0.443 ± 0.008 mm *vs.* 0.525 ± 0.005 mm) (Fig. 7A). The EVC control demonstrated a similar trend (0.335 ± 0.01mm *vs.* 0.460 ± 0.016 mm) (Fig. 7B). The secretion of TNF-α, IL-1β and TNF-α/IL-1β clones had reduced angiogenic potential in normoxia and hypoxia compared to the relevant EVC (Fig. 7C/D). In normoxia, this reached significance for TNF-α (p < 0.01), IL-1β (p < 0.01) and TNF-α/IL-1β (p < 0.05) compared with EVC (Fig. 7C). In hypoxia, the reduction in angiogenic potential was significant in TNF-α/IL-1β compared to EVC (p < 0.01) (Fig. 7D). These results suggest that the HBEC4 ctl and EVC secrete different angiogenic mediators depending on oxygen concentration, and that TNF-α and IL-1β are produced at a level, which promotes an anti-angiogenic response.

## Discussion

This project employed a normal bronchial epithelial cell line (HBEC4), immortalised in the absence of viral onco-proteins^32^. The purpose was to establish a new inflammatory bronchial epithelia cell model for the use in the study of inflammatory mediators in the process of lung carcinogenesis. TNF-α and IL-1β were overexpressed alone or in combination and cells were cultured under normoxic and hypoxic (0.5% oxygen) conditions. The aim was to characterise genotypic and phenotypic alterations over time, with the objective of promoting neoplastic transformation.

qPCR arrays (Fig. 2) demonstrated a large number of gene changes in all clones under both normoxic and hypoxic conditions in pathways associated with apoptosis, invasion and metastases, proliferation and angiogenesis. Under normoxia, MMP1 and integrin (ITGA4) were elevated in TNF-α and TNFα/IL-1β clones; these can degrade the ECM component and play a role in the development of tumour metastasis^39^. In the hypoxic clones, an increase was also observed in the MMP gene family, which are critical in hypoxia mediated invasion of lung adenocarcinoma^40^. In the hypoxic clones, integrin expression was up-regulated; these genes are critical as they can mediate signals that induce gene expression, resulting in cell adhesion and tumour growth^39^. MMP2 and MMP9 are associated with lung cancer and can be secreted by cancer cells resulting in invasion and metastasis^41^. *CXCL8/IL8* levels were also increased in the normoxic and hypoxic clones and it has been determined that high levels of CXCL8 are associated with both current and subsequent diagnosis of lung cancer (up to 2 years) and is significantly increased in the serum of lung cancer patients compared with controls^42^. As *c-Myc* and *p53* were part of a five-gene change which conferred HBEC malignancy^34^, these were also examined by qPCR. Levels of *c-Myc* were increased in the normoxic and hypoxic overexpression clones (Supplementary Fig. 2), while little change was observed in *p53*.

A number of assays were carried out to validate gene changes in a functional manner. The proliferative rate of the cells significantly increased under both normoxia and hypoxia, after three months of culture, this included the HBEC4 ctl under hypoxia (Fig. 3).

A qualitative cell migration assay was used to determine if the migration capacity of the cells had modified. All scratches were completely closed in all clones at 24 h under normoxia (Fig. 4). In hypoxia, the wound remained unclosed in all clones even after 24 h of culture (Fig. 4). There are a number of reasons as to why this might have happened including alterations in genes involved in EMT such as *MMP2, 9*, *MTA1* and *TWIST1* (Fig. 2). Hypoxia occurs in the central region of a tumour, where tumour and immune cells tend to have less migratory capacity, for example the inhibition of chemotaxis under hypoxia, through the up-regulation of MAPK phosphatase 1 (MKP-1), which may account for the accumulation of macrophages in hypoxic regions of tumours^43^. However, normoxic cells demonstrated a greater migration capacity, which mimics the environment at the leading edge of tumours and can drive tumour metastases. It is also possible that the rate of migration was affected by the proliferative rate of the cells, which was different between the same clones under hypoxia and normoxia. Other studies have demonstrated decreased cellular migration capacity under hypoxia^44^,^45^,^46^.

Adhesion molecules are also important in terms of migration capacity, such as ICAM-1 and VCAM-1 (Fig. 5). Both have been implicated in cancer^47^ and can be regulated by TNF-α and IL-1β^38^. There was a substantial increase in ICAM-1 in the IL-1β clone compared with EVC under normoxia. Indeed, levels where higher in the IL-1β clone compared with the dual expressing clone (TNF-α and IL-1β). Other studies have shown that IL-1β has a more potent effect on ICAM-1 than TNF-α^38^,^48^. In addition, the expression of NF-κB (NFKB1) may have provided a confounding effect on the expression of these molecules as it can not only regulate TNF-α and IL-1β but can also promote the expression of ICAM and VCAM^49^. As shown in Fig. 2 *NFKB1* was elevated in all overexpression clones under normoxia (relative to the EVC), while this was only evident in the IL-1β clone in hypoxia. Hypoxia stimulated an up-regulation of ICAM-1 in both the HBEC4 ctl and EVC. The low level of VCAM-1 may be accounted for as it is expressed predominantly on endothelial cells, while epithelial cells predominantly express ICAM.

The invasive capacity of the clones under normoxia and hypoxia was examined (Fig. 6). Hypoxia promoted a more invasive phenotype in both the HBEC4 ctl and EVC. Under normoxia, the TNFα/IL-1β clone had a significant increase in invasive capacity compared with EVC. IL-1β can increase cellular invasive capacity^50^, as well as TNF-α^51^. Under hypoxia, no difference in the invasive capacity was evident in the overexpression clones compared with EVC. It would appear that it is hypoxia itself, which is the more important factor in invasion. Hypoxia has been linked to invasion^52^ and cancer progression^53^. In addition, the increased levels of *S100A4* in the hypoxic HBEC4 ctl/EVC and the normoxic TNF-α/IL-1β clone may have aided invasion. *S100A4* has a role in tumour progression and metastasis and is involved in NSCLC^54^ metastases. Also, IL-1β can promote metastases, via induction of adhesion molecules such as ICAM and VCAM^55^,^56^, which are up regulated in the clones (Fig. 5A/B).

Lung cancer is an angiogenic dependent cancer and one of the important mediators, VEGF, has been shown to be an autocrine growth factor in NSCLC^57^. An endothelial tube formation assay was performed using Ea.hy926 cells seeded in supernatants collected from over-expression clones (Fig. 7). Tube lengths were decreased in supernatants collected from EVC/HBEC4 under hypoxia compared to EVC/HBEC4 under normoxia. The tube lengths were also significantly decreased in supernatants collected from TNF-α, IL-1β and TNF-α/IL-1β clones relative to EVC under normoxia. TNF-α and IL-1β can promote angiogenesis, through induction of VEGF and CXCL8^58^,^59^, however, this is dependent on the cytokine concentration. TNF-α, at low concentrations, promotes vascularisation but, at high concentrations, causes vessel destruction^60^. It is interesting to note that *VEGF* levels are decreased in the over expression clones at both normoxia and hypoxia (Fig. 2). The cytokine balance is critical for angiogenesis^61^,^62^ and it may be that the levels of cytokine secretion in clones were not elevated sufficiently to stimulate a more angiogenic phenotype. Other studies have demonstrated that these cytokines can stimulate both pro and anti oncogenic effects, which are environment dependant^25^,^61^,^63^,^64^.

The development of anchorage independent growth is an important indicator of a neoplastic state. Soft agar assays were performed with overexpressing clones from both normoxic and hypoxic conditions, however anchorage independent growth was not evident (Data not shown). The mutational status of KRAS^v12^ was also screened, as this is a common mutation in NSCLC^65^ and is linked to an inflammatory response^66^. No mutations were detected in any of the clones under either normoxic or hypoxic conditions (Data not shown).

The interplay between hypoxia and inflammation is complex and is considerably driven through NF-κB regulated and responsive mechanisms^27^,^28^. In addition, this transcription factor can control the expression of cytokines. The link between HIF-1α and inflammation has been established and some studies have shown that this factor is up regulated under normoxic conditions through inflammatory mediators such as TNF-α and IL-1β^67^,^68^,^69^. These multifaceted relationships and interactions may perhaps have influenced the effect of the overexpression of TNF-α and IL-1β alone and in combination in the HBEC4 cells and differential cellular responses such as adhesion, invasion and angiogenic potential.

At the end of the three months the mRNA and protein levels of TNF-α and IL-1β were determined. While TNF-α protein levels remained constant, the IL-1β levels had reduced to that of the EVC level in the TNFα/IL-1β clone. At the mRNA level, TNF-α was robustly expressed, however expression was decreased under hypoxia. IL-1β expression, like TNF-α, was reduced under hypoxia. Although the mRNA levels were similar in both clones (IL-1β *vs.* TNF-α/IL-1β), the protein levels were lower and this could be a consequence of epigenetic or miRNA regulatory mechanisms. As levels of IL-1β were low, its expression was examined in genomic DNA samples. IL-1β was not detected in the TNF-α/IL-1β clone, which would suggest that a recombination event had occurred within the gene. Another potential issue may be that the integration of IL-1β in the cells occurred at the TNF-α locus. This would mean that two copies of IL-1β were subsequently present i.e. native gene versus construct. Therefore the presence of TNF-α from the integrated construct would still be able to induce IL-1β from the extant native gene(s). As a last potential confounding issue, because we believe that IL-1β expression was lost from the clone, this may have been due to a deletion event in the integrated genomic region, which may have left the constitutive promoter driving its expression continuously active. This in itself could potentially titrate out transcription factors leading to alterations in gene expression.

However, as the expression of IL-1β was high in the second month of the experiment, and in that time may have aided in molecular and cellular changes within the clone, and as it has also been shown that only small amounts of IL-1β are required for an effect^70^, the TNFα/IL-1β clone was used in the end point experiments. Differences were detected in a number of gene and cell based assays between the dual and single expressing cytokine clones, which further strengthens that, the expression of IL-1β had induced changes while it was expressed. In addition, it may have prompted irreversible epigenetic change, resulting in the constitutive activation of a number of IL-1β responsive pathways.

In future studies, TNF-α and IL-1β protein could be isolated and quantified from these cell lines and used as conditioned media in which to culture un-transfected HBEC4 cells, thus ruling out any issues which may arise from integration or recombination events. This would also allow for increased periods of continuous culture.

The data generated in this study provides support that TNF-α, IL-1β and hypoxia promote a neoplastic phenotype in normal bronchial epithelial cells, after only three months of culture. This was through the modification of gene expression profiles in a number of hallmark cancer pathways and the amplification of a number of critical cellular functions such as proliferation, invasion, adhesion and migration. As pro-longed chronic exposure to inflammation is a pre-requisite for many disease states, these results warrant extended growth studies to further delineate the complex roles of TNF-α, IL-1β and hypoxia in the lung carcinogenesis. We believe that extended culture will induce further oncogenic changes and the development of anchorage independent growth, which in turn will dictate the need for an orthotopic model in nude mice.

This project provides an essential inflammatory *in vitro* model for further immuno-oncology studies in the lung cancer setting and may provide benefit for biomarker and/or immunotherapy targeted studies.

## Methods

### Cell Culture

HBEC4 cells were maintained in keratinocyte serum-free media (SFM), with L-glutamine (GIBCO Invitrogen, Paisley, Scotland) and supplemented with human recombinant epidermal growth factor (rEGF) and bovine pituitary extract (GIBCO Invitrogen). The HBEC4 cell line was a kind gift from Prof. John Minna, Hamon Centre for Therapeutic Oncology Research, UTSouthwestern, Dallas, TX, USA. The Ea.hy926 cell line (ATCC, LGC Promochem, Teddington, UK) was cultured in DMEM, supplemented with 10% (v/v) FBS, penicillin streptomycin (500 U/mL) and HAT (100 μM hypoxanthine, 0.4 μM aminopterin, 16 μM thymidine). All cell lines were maintained at 37 °C in a humidified atmosphere with 5% CO_2_. Plastics used with HBEC4 were coated in collagen (0.5 g/L dH_2_O; Gelatin from porcine skin-type A, Sigma, St. Louis, MO, US).

### Hypoxic conditions

Cells were cultured in an Invivo_2_ 400 Hypoxia Workstation with Ruskinn hypoxia gas mixer (Biotrace Int Plc, Glamorgen, UK) with the oxygen concentration set at 0.5%. Hypoxic conditions in cell supernatants were confirmed by pO_2_ measurements taken using a pO2 E series sensor (BF/OT/E) and an OxyLab pO2™ (Oxford Optronix Ltd, Oxford, UK).

### Stable over-expression of TNF-α and IL-1β

All media and reagents, including vectors, were purchased from InvivoGen (San Diego, CA, USA). The TNF-α (pORF-hTNFa v12) and IL-1β (pCLEF30-hIL01B v15) plasmids were sub-cloned into a pVITRO1-*mcs* vector, alone or in combination. An empty vector control (EVC) was also included. Plasmids were stably transfected into HBEC4 using FuGENE® 6 (Roche Diagnostics GmbH, Mannheim, Germany) according to manufacturer’s instructions. A mixed population of stable clones were isolated, expanded and selected in hygromycin (50 μg). Four cell sub-lines were developed (i) EVC, (ii) TNF-α alone, (iii) IL-1β alone and (iv) TNF-α and IL-1β combined.

### Functional confirmation of TNF-α and IL-1β clones

Inserts were confirmed using sequencing (3130xl genetic analyser, ABI Biosystems, CA, USA in conjunction with BioEdit v 7.0.8 software, Tom Hall, Ibis Biosciences, CA, USA) and by RT-PCR for the antibiotic selection marker, hygromycin (Data not shown). TNF-α (710 bp; Fwd: ATGAGCACTGAAAGCATGATC, Rev: TCACAGGGCAATGATCCCAAAG) and IL-1β (816 bp; Fwd: GCAGCCATGGCAGAAGTACCTGAGCTC, Rev: TTAGGAAGACACAAATTGC A) expression was confirmed by RT-PCR. CXCL8 was included as a positive control (297 bp; FWD: ATGACTTCCAAGCTGGCCGTG, Rev: TGAATTCTCAAGCCCTCTTCA) and beta-actin as a loading control (560 bp: Fwd: TGTTTGAGACCTTCAACACCC, Rev: AGCACTGTGTTGGCGTACAG). A standard protocol using 35 cycles (G Storm thermal cycler, GSI, Braintree, Essex, UK) was used with a target annealing temperature of 55 °C for all primers.

TNF-α and IL-1β protein secretion was confirmed by ELISA according to the manufacturer’s instructions (Duo Set ELISA, R and D Systems, Minneapolis, MN, USA). The substrate solution was phosphate citrate buffer (containing citric acid and disodium hydrogen orthophosphate dodecahydrate pH 5.0) with 10 mg of 1,2-phenylenediamine dihydrochloride (OPD) and 18 μL 1 M H_2_O_2_. One hundred μL of substrate solution was added to each well and reaction stopped with the addition of 50 μL 1M H_2_SO_4_. Absorbance was read at 492 nm with cytokine concentration determined by interpolating from a standard curve of known concentrations for each cytokine (Data not shown).

### The Human Cancer PathwayFinder™ RT^2^ Profiler

RNA was prepared using the Qiagen RNeasy® Mini Kit with the on-column DNase treatment step as per manufacturer’s instructions. A reverse-transcription reaction was carried out using an RT^2^ First Strand Kit (SA Biosciences, Qiagen Inc., Valencia, CA, USA) to generate cDNA from 1 μg of RNA. A qPCR master mix was prepared and added to the arrays (SA Biosciences) according to manufacturer’s instructions. Real-time PCR detection was performed on an ABI Prism 7500 (ABI Biosystems, Foster City, CA, USA). The threshold cycle (Ct), for each well was calculated using the instrument software. Data analysis was carried out using an online-based analysis template, based on the 2^ΔΔCt^ method with raw data being normalised to the housekeeping genes on the array plate (http://www.sabiosciences.com). qPCR primers were purchased from Qiagen for the validation of specific genes (TP53 (p53), S100A4, DR3 (TNFRSF25) and c-Myc).

### Cellular proliferation assays

Cellular proliferation was measured using a Cell Proliferation ELISA, BrdU (Roche Diagnostics Ltd., Sussex, UK) according to the manufacturer’s instructions. Briefly, cells were seeded at 5 × 10^3^/well in a 96-well plate, adhered overnight and cultured for a period of 24–48 h. Absorbance was measured on a plate reader at 450 nm with a reference wavelength set to 690 nm. Wells containing EVC cells were used for normalisation purposes and set to 100%.

### Migration capacity

Cells were seeded in a six well plate at 5 × 10^4 ^cells/well and cultured until 100% confluent. Reference markings were made on the outside of the bottom of the well, for reference points when obtaining images. The cell monolayer was then scraped once using a sterile p200 pipette tip. The media was removed and cells washed in PBS to smooth the edge of the scratch and fresh media added. The scratches were photographed at the beginning (Time 0) and 24 h (Time 24) post scratch. Care was taken to ensure that the scratches were photographed in approximately the same field at each time point.

### ICAM and VCAM determination by flow cytometry

Cells were seeded in a six well plate at 5 × 10^4 ^cells/well and pelleted after 96 h of culture. The cell pellet was washed twice with 1 mL FACS buffer (2% FBS, 0.1% Sodium Azide in PBS). The wash was removed and 500 μL of FACS buffer and 500 μL of FBS were used to re-suspend the pellet. The samples were incubated at RT for 5 min and a further 1 mL FACS buffer added to the tube. The cells were centrifuged at 1500 χ g for 3 min and the supernatant discarded. Two μL of appropriate antibody, anti-ICAM-1 or anti-VCAM-1 (FITC labelled; R & D systems), or isotype control (FITC labelled IgG2A; R & D systems) was added to the appropriate tube and vortexed. After incubating in the dark at 4 °C for 30 min, 1 mL of FACS buffer was added to the tubes and vortexed briefly. The tubes were centrifuged as above and the supernatant discarded. The pellet was re-suspended in 500 μL of FACS buffer and stored in the dark at 4 °C for 30 min. The surface levels of ICAM-1 and VCAM-1 were measured using a FACS Caliber flow cytometer (BD Biosciences. San Jose, CA, USA). Data analysis was carried out using dot plot analysis.

### Invasion assay

The invasive capacity of cells was examined using a florescent-based Chemicon 96-well Cell Invasion Assay according to manufacturer’s instructions (Millipore, Billerica, MA, USA). The chemo-attractant used was complete media supplemented with 10% FBS. Intensity was measured using a fluorescence plate reader at the 480/520 nm filter setting. Wells without cells but containing Cell Detachment Buffer, Lysis Buffer, and CyQuant Dye were used as blanks. The fluorescence values from these wells were subtracted from all other values in order to interpret the data. Values were compared to the EVC or normoxia (as appropriate) and set to 100%.

### Endothelial tube formation assay

The endothelial tube formation assay was carried out as follows: Matrigel (Becton, Dickinson, Franklin Lakes, NJ, USA) was thawed on ice overnight at 4 °C. Fifty μL of Matrigel was added to each well in a 96-well plate and solidified over 30 min at 37 °C. The Ea.hy926 cells were re-suspended at 3 × 10^4 ^cells/mL in supernatant collected from the various cell sub-lines (EVC, TNF-α, IL-1β, TNF-α/IL-1β) and HBEC4 ctl under both normoxic and hypoxic conditions and pipetted on top of the Matrigel layer. Cells were incubated at 37 °C for 16 h and endothelial tube lengths were measured using Image J software from the NIH (http://rsb.info.nih.gov/ij/).

### Statistical analysis

The data are expressed as mean ± SEM. Statistical analysis was performed with Graphpad Prism 5.01 (Graphpad Software, La Jolla, CA, USA) using either a paired student’s *t*-test (groups of two) or a one-way analysis of variance (ANOVA) (three or more groups). Following ANOVA a *post hoc* test was performed as indicated in the figure legends. Differences were considered significant when *p* < 0.05.

## Additional Information

**How to cite this article**: Baird, A.-M. *et al.* Promotion of a cancer-like phenotype, through chronic exposure to inflammatory cytokines and hypoxia in a bronchial epithelial cell line model. *Sci. Rep.* **6**, 18907; doi: 10.1038/srep18907 (2016).

## Supplementary Material

Supplementary Figures
