# Supplementary material for: Promotion of a cancer-like phenotype, through chronic exposure to inflammatory cytokines and hypoxia in a bronchial epithelial cell line model
Source: Sci Rep. 2016 Jan 13;6:18907. doi: 10.1038/srep18907 (PMC4725362; doi:10.1038/srep18907)
Supplement: Supplementary Figures [file srep18907-s1.pdf]

Promotion of a cancer-like phenotype, through chronic exposure to inflammatory cytokines and hypoxia in a bronchial epithelial cell line model

Anne-Marie Baird[1, 2], Steven G. Gray[2, 3], Derek J. Richard[1], Kenneth J. O'Byrne* [1, 2,4]

[1]Cancer and Ageing Research Program, Queensland University of Technology, Brisbane, Australia

[2]Thoracic Oncology Research Group, Institute of Molecular Medicine, Trinity College Dublin, Ireland

[3]HOPE Directorate, St. James's Hospital, Dublin 8, Ireland

[4]Division of Cancer Services, Princess Alexandra Hospital, Brisbane, Australia

***Corresponding author for proof and reprints:**

Prof. Kenneth J. O'Byrne, Cancer and Ageing Research Program, Queensland University of Technology, Institute of Health and Biomedical Innovation, TRI Level 3, 37 Kent St, Woolloongabba QLD 4102, Australia

Tel: +61-7-3176 6505

E-mail: kenneth.o'byrne@health.qld.gov.au

A.

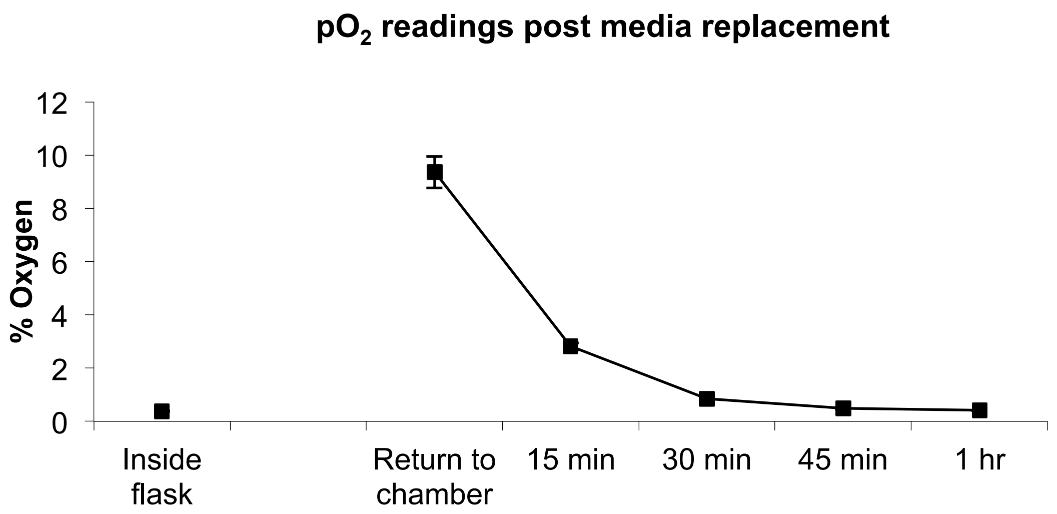

B.

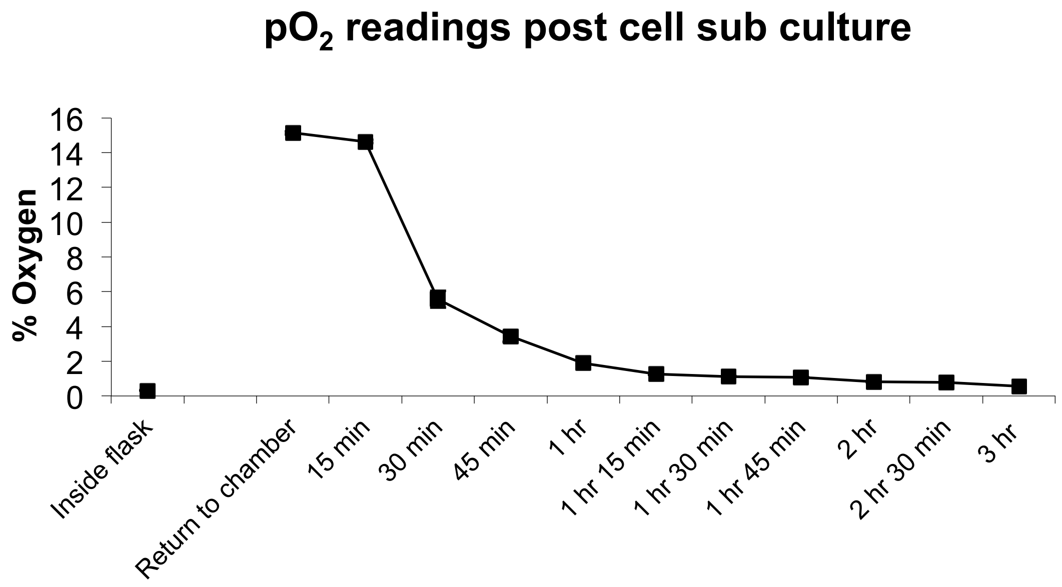

Fig S1: Under hypoxia, media was changed and cell sub-culturing was performed under ambient oxygen. Media replacement was performed within 15 min every 4 days and sub-culturing within 60 min on reaching 70% confluency. To confirm cells were experiencing hypoxic conditions (chamber set at 0.5% O_2), and also to determine the percentage of re-oxygenation occurring during media replacement and sub-culture, pO_2 measurements were recorded by means of a pO_2 probe (pO2 E series sensor (BF/OT/E) Oxford Optronix Ltd, Oxford, UK) and an OxyLab pO2™ (Oxford Optronix Ltd). Cells were discarded after the experiment due to non-sterile conditions

within the hypoxia chamber. Readings were obtained in mm/Hg and converted into oxygen percentages using the following relationship: mHg value/0.76 x 0.1, where 0.76 mmHg is approximately equivalent to 0.1% oxygen (Personal communication – Dr. Laure Marignol, Dept. of Radiation Therapy and Prostate Cancer Research Group, Trinity College Dublin). Data is graphed as mean ± SEM
(n=3).

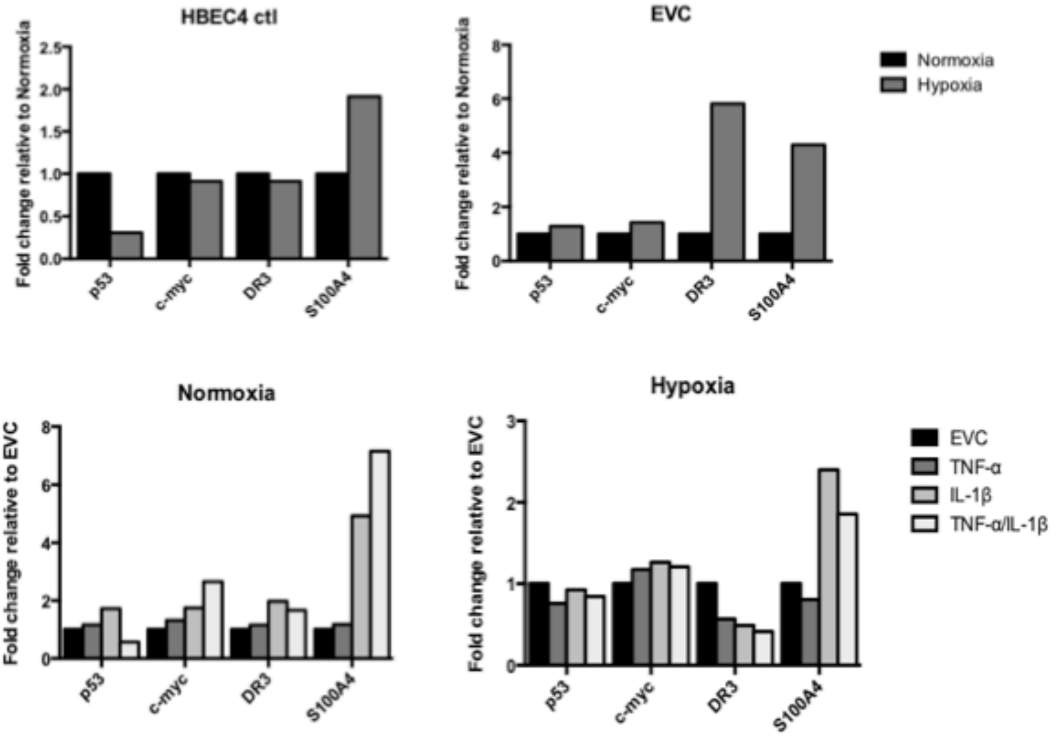

Fig S2: A selection of genes was also examined using qPCR primer sets. Results are graphed as fold changes compared with appropriate EVC. Data was analysed based on the $2^{\Delta\Delta ct}$ method with samples normalised to 18S control values. (n=1)
